# Supplementary material for: SARS-CoV-2 specific antibody and neutralization assays reveal the wide range of the humoral immune response to virus
Source: Commun Biol. 2021 Jan 29;4:129. doi: 10.1038/s42003-021-01649-6 (PMC7846565; doi:10.1038/s42003-021-01649-6)
Supplement: Supplementary file 2 — Supplementary Information [file 42003_2021_1649_MOESM2_ESM.pdf]

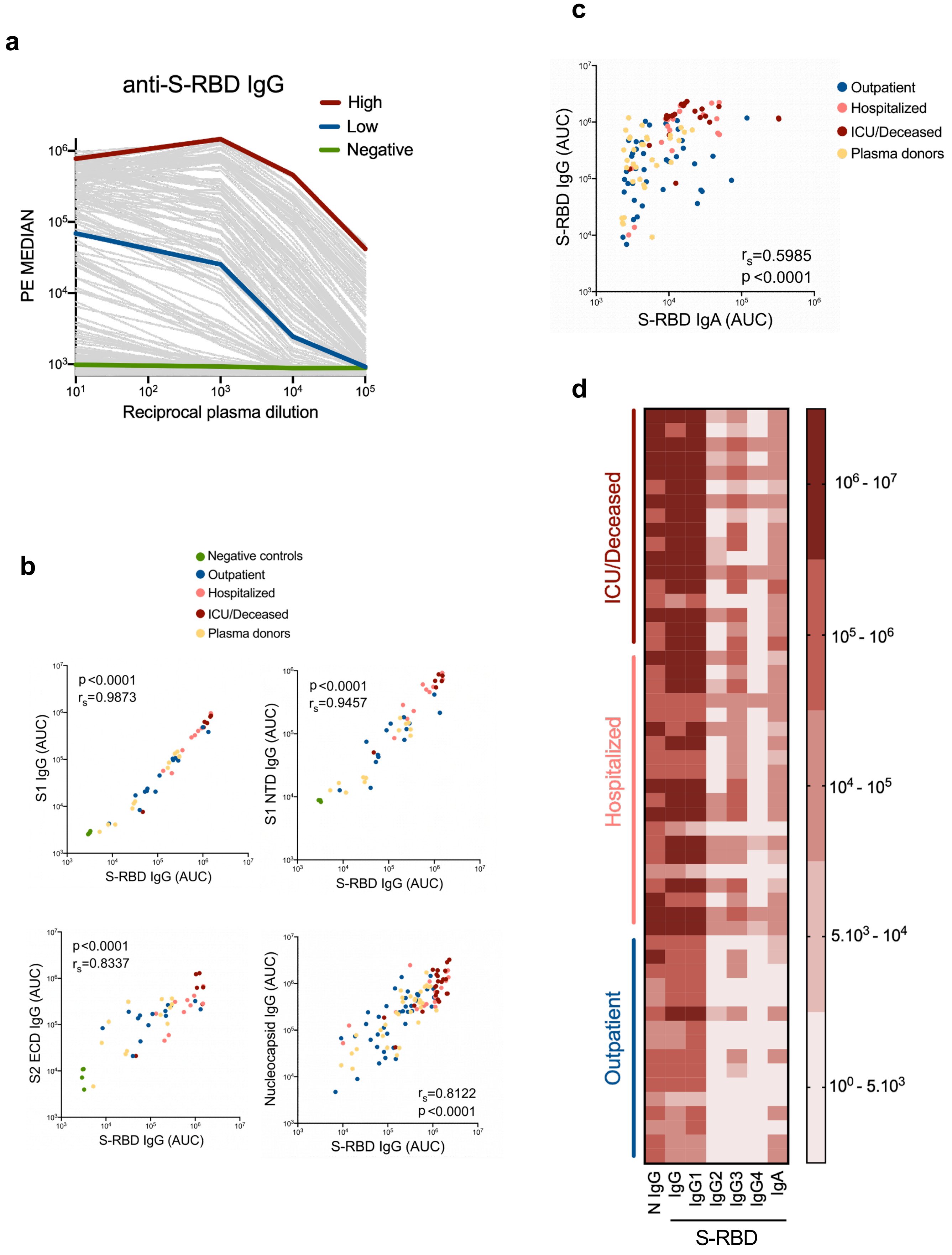
a
anti-S-RBD IgG
High
Low
Negative
PE MEDIAN
Reciprocal plasma dilution
b
Negative controls
Outpatient
Hospitalized
ICU/Deceased
Plasma donors
p<0.0001
rs=0.9873
S1 IgG (AUC)
S-RBD IgG (AUC)
p<0.0001
rs=0.9457
S1 NTD IgG (AUC)
p<0.0001
rs=0.8337
S2 ECD IgG (AUC)
rs=0.8122
p<0.0001
Nucleocapsid IgG (AUC)
c
Outpatient
Hospitalized
ICU/Deceased
Plasma donors
S-RBD IgG (AUC)
rs=0.5985
p<0.0001
S-RBD IgA (AUC)
d
ICU/Deceased
Hospitalized
Outpatient
N IgG
IgG
IgG1
IgG2
IgG3
IgG4
IgA
S-RBD
10^6 - 10^7
10^5 - 10^6
10^4 - 10^5
5.10^3 - 10^4
10^0 - 5.10^3

Supplementary Figure 1: SARS-CoV-2 S-RBD antibody detection

a. Antibody assay measuring the plasma reactivity to S-RBD. Flow cytometry analysis of the PE fluorescence conjugated to anti-human IgG recognizing antibodies, present in the patient plasma and bound to S-RBD protein on the beads. Means of PE values in reciprocal dilutions were used to generate a curve for each positive plasma. Subject plasma with high and low antibody levels and a healthy control plasma were color-coded as red, blue and green, respectively. **b.** Correlation of S1 subunit IgG (n=46), S1 N Terminal Domain (NTD) IgG (n=46), S2 Extracellular Domain (ECD) IgG (n=46) and Nucleocapsid IgG with S-RBD IgG (n=115). **c**. Correlation of S-RBD IgA with S-RBD IgG (n=115). Two-tailed Spearman's was used to determine statistical significances. **d**. Heat map represents AUC values of Nucleocapsid (N) protein IgG, S-RBD IgG, S-RBD IgG subclasses and S-RBD IgA antibodies from individual subjects clustered as outpatients, hospitalized and ICU or deceased.

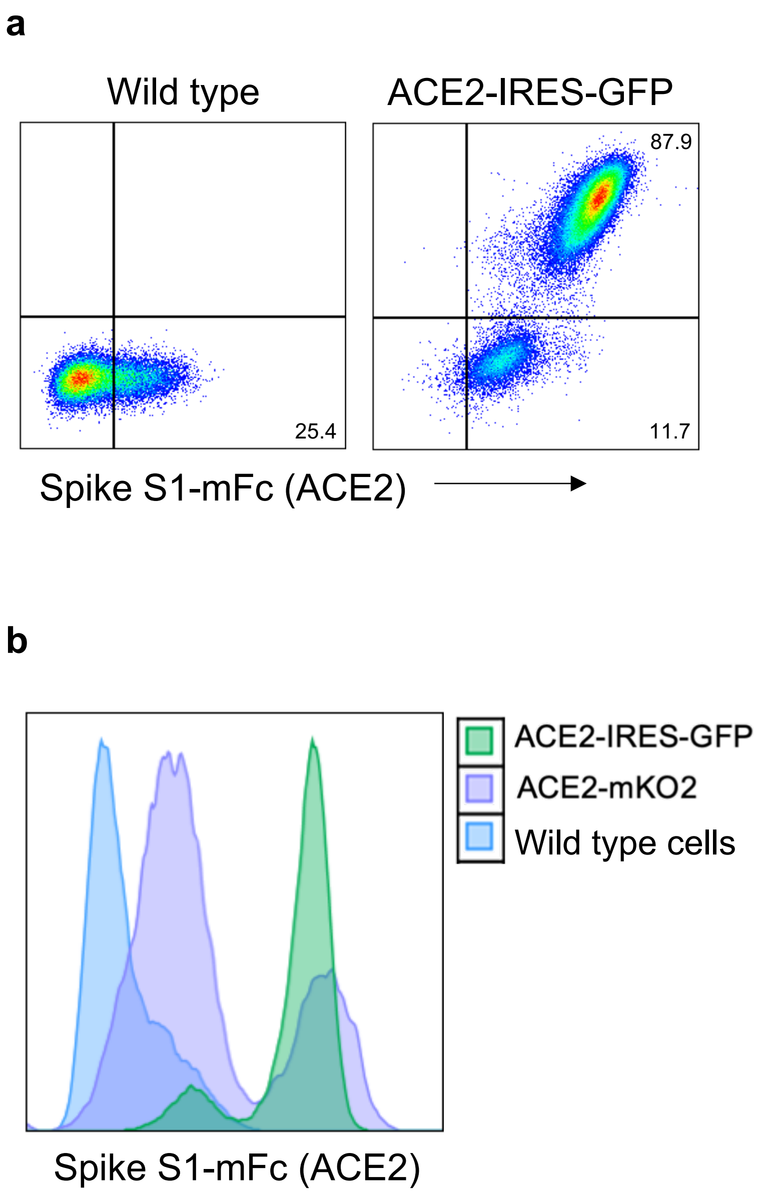
a
Wild type
ACE2-IRES-GFP
87.9
25.4
11.7
Spike S1-mFc (ACE2)
b
ACE2-IRES-GFP
ACE2-mKO2
Wild type cells
Spike S1-mFc (ACE2)

Supplementary Figure 2: ACE2 detection on cell surface membrane

a. Wild type or ACE2-IRES-GFP over-expressing 293 cells were stained with SARS-CoV-2 S1 protein fused to mouse Fc, and anti-mouse Fc secondary antibody. **b**. ACE2 expression, detected as in **a**, in wild type and ACE2 overexpressing 293 cells compared in an overlay of flow data.

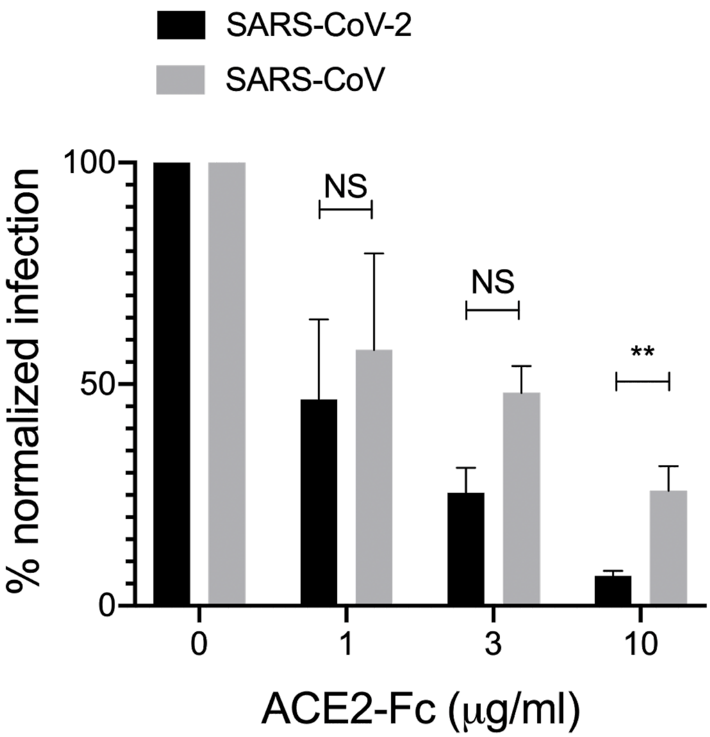
SARS-CoV-2
SARS-CoV
100
50
0
% normalized infection
NS
NS
**
0
1
3
10
ACE2-Fc (μg/ml)

Supplementary Figure 3: Neutralization of SARS-CoV-2 and SARS-CoV pseudoviruses

a. Normalized percent infection levels of SARS-CoV-2 and SARS-CoV pseudoviruses in neutralization assay using soluble ACE2 at 1 mg/mL, 3 mg/mL and 10 mg/mL concentrations. Gray and black columns show SARS-CoV and SARS-CoV-2 data, respectively. Graphs represent 5 replicates of experiments with 1 mg/mL and 10 mg/mL and 3 replicates with 3 mg/mL concentration. Significance was determined using two-tailed Mann-Whitney U test.

a

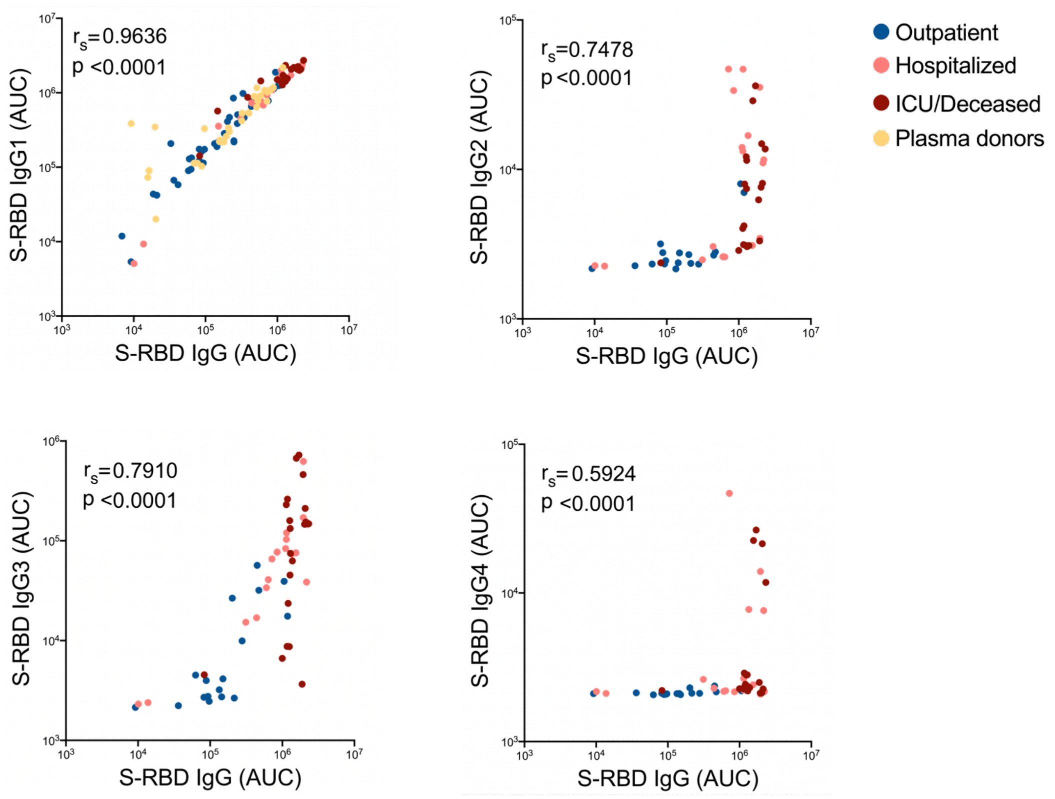

r_s=0.9636
p <0.0001
S-RBD IgG1 (AUC)
S-RBD IgG (AUC)
r_s=0.7478
p <0.0001
S-RBD IgG2 (AUC)
S-RBD IgG (AUC)
Outpatient
Hospitalized
ICU/Deceased
Plasma donors
r_s=0.7910
p <0.0001
S-RBD IgG3 (AUC)
S-RBD IgG (AUC)
r_s= 0.5924
p <0.0001
S-RBD IgG4 (AUC)
S-RBD IgG (AUC)

b

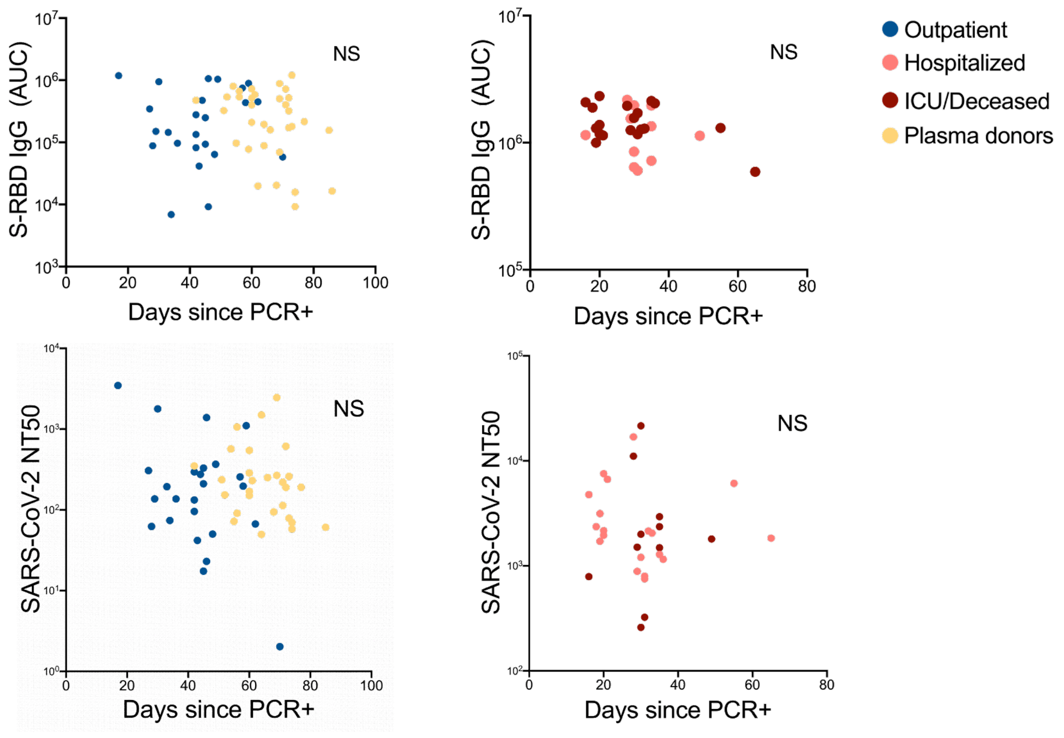

S-RBD IgG (AUC)
NS
Days since PCR+
S-RBD IgG (AUC)
NS
Days since PCR+
Outpatient
Hospitalized
ICU/Deceased
Plasma donors
SARS-CoV-2 NT50
NS
Days since PCR+
SARS-CoV-2 NT50
NS
Days since PCR+

Supplementary Figure 4: S-RBD IgG subclasses correlation with total S-RBD IgG

a. Correlation of AUC levels of S-RBD specific IgG subclasses (IgG1, 2, 3 and 4) with S-RBD specific total IgG (n= 115, 74, 74 and 74, respectively). **b**. Correlation graphs of S-RBD IgG and NT50 with the number of days between PCR confirmation and the blood draw. Two-tailed Spearman's was used to determine the statistical significance.

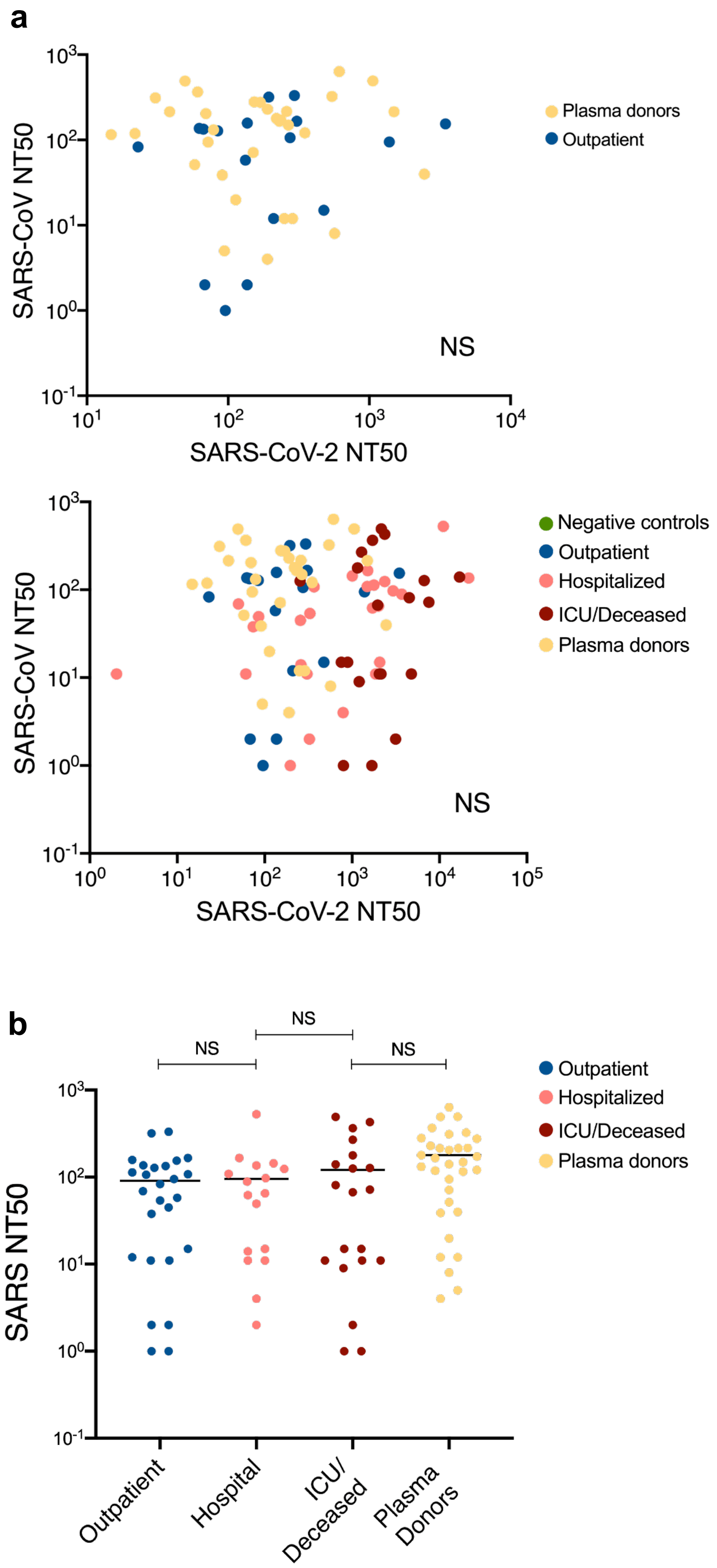
a
SARS-CoV NT50
SARS-CoV-2 NT50
Plasma donors
Outpatient
NS
Negative controls
Outpatient
Hospitalized
ICU/Deceased
Plasma donors
NS
b
NS
NS
NS
SARS NT50
Outpatient
Hospital
ICU/ Deceased
Plasma Donors
Outpatient
Hospitalized
ICU/Deceased
Plasma donors

Supplementary Figure 5: SARS-CoV-2 NT50 correlation with SARS-CoV NT50

a. Correlation of SARS-CoV-2 NT50 with SARS-CoV when all severity groups were combined, or only outpatient and plasma donor subjects were combined. **b**. SARS-CoV neutralization titers (NT50) of COVID-19 plasma grouped as outpatient, hospitalized, ICU or deceased and convalescent plasma donor groups. Two-tailed Spearman's was used for statistical analysis.
