# Supplementary material for: SARS-CoV-2 specific antibody and neutralization assays reveal the wide range of the humoral immune response to virus
Source: Commun Biol. 2021 Jan 29;4:129. doi: 10.1038/s42003-021-01649-6 (PMC7846565; doi:10.1038/s42003-021-01649-6)
Supplement: Supplementary file 3 — Description of Additional Supplementary Files [file 42003_2021_1649_MOESM3_ESM.pdf]

## Description of Additional Supplementary Files

**File name:** Supplementary Data 1

**Description:** Source Data for patient demographics and clinical characteristics, pseudovirus titrations and neutralization assay graphs. Subject IDs, patient demographics, measured antibody levels and neutralization titers data were provided in “master table” sheet. Other source data were provided in the sheets corresponding to their figures.

*ICU = Intensive care unit.
