# Supplementary material for: SARS-CoV-2 specific antibody and neutralization assays reveal the wide range of the humoral immune response to virus
Source: Commun Biol. 2021 Jan 29;4:129. doi: 10.1038/s42003-021-01649-6 (PMC7846565; doi:10.1038/s42003-021-01649-6)
Supplement: Supplementary file 5 — Reporting Summary [file 42003_2021_1649_MOESM5_ESM.pdf]

nature research

Corresponding author(s): Derya Unutmaz

Last updated by author(s): Dec 8, 2020

Reporting Summary

Nature Research wishes to improve the reproducibility of the work that we publish. This form provides structure for consistency and transparency in reporting. For further information on Nature Research policies, see our Editorial Policies and the Editorial Policy Checklist.

Statistics

For all statistical analyses, confirm that the following items are present in the figure legend, table legend, main text, or Methods section.

n/a	Confirmed	
☐	☒	The exact sample size (n) for each experimental group/condition, given as a discrete number and unit of measurement
☐	☒	A statement on whether measurements were taken from distinct samples or whether the same sample was measured repeatedly
☐	☒	The statistical test(s) used AND whether they are one- or two-sided<br>*Only common tests should be described solely by name; describe more complex techniques in the Methods section.*
☒	☐	A description of all covariates tested
☒	☐	A description of any assumptions or corrections, such as tests of normality and adjustment for multiple comparisons
☒	☐	A full description of the statistical parameters including central tendency (e.g. means) or other basic estimates (e.g. regression coefficient) AND variation (e.g. standard deviation) or associated estimates of uncertainty (e.g. confidence intervals)
☐	☒	For null hypothesis testing, the test statistic (e.g. F, t, r) with confidence intervals, effect sizes, degrees of freedom and P value noted<br>*Give P values as exact values whenever suitable.*
☒	☐	For Bayesian analysis, information on the choice of priors and Markov chain Monte Carlo settings
☒	☐	For hierarchical and complex designs, identification of the appropriate level for tests and full reporting of outcomes
☒	☐	Estimates of effect sizes (e.g. Cohen's d, Pearson's r), indicating how they were calculated

Our web collection on statistics for biologists contains articles on many of the points above.

Software and code

Policy information about availability of computer code

Data collection	Microsoft Excel 16.36 for clinical data collection and management; BD FACSDiva Software Version 8.0.2 for flow sorting.
Data analysis	FlowJo 10.6.2 for FACS analysis; Microsoft Excel 16.36; GraphPad Prism 8.4.3 for data organization and graphical demonstration

For manuscripts utilizing custom algorithms or software that are central to the research but not yet described in published literature, software must be made available to editors and reviewers. We strongly encourage code deposition in a community repository (e.g. GitHub). See the Nature Research guidelines for submitting code & software for further information.

Data

Policy information about availability of data

All manuscripts must include a data availability statement. This statement should provide the following information, where applicable:

- Accession codes, unique identifiers, or web links for publicly available datasets
- A list of figures that have associated raw data
- A description of any restrictions on data availability

The source data for the Figures along with the Supplementary Figures presented in this paper are available as Supplementary Data 1

Field-specific reporting

Please select the one below that is the best fit for your research. If you are not sure, read the appropriate sections before making your selection.

☒ Life sciences ☐ Behavioural & social sciences ☐ Ecological, evolutionary & environmental sciences

For a reference copy of the document with all sections, see nature.com/documents/nr-reporting-summary-flat.pdf

Life sciences study design

All studies must disclose on these points even when the disclosure is negative.

Sample size	Sample size of 115 individuals was based on how many we were able to recruit for blood donations.
Data exclusions	No data were excluded.
Replication	All experiments successfully repeated at least twice.
Randomization	This is not relevant as this is an observational study.
Blinding	This is not relevant as this is an observational study.

Reporting for specific materials, systems and methods

We require information from authors about some types of materials, experimental systems and methods used in many studies. Here, indicate whether each material, system or method listed is relevant to your study. If you are not sure if a list item applies to your research, read the appropriate section before selecting a response.

Materials & experimental systems

n/a	Involved in the study	
☐	☒	Antibodies
☐	☒	Eukaryotic cell lines
☒	☐	Palaeontology and archaeology
☒	☐	Animals and other organisms
☐	☒	Human research participants
☒	☐	Clinical data
☒	☐	Dual use research of concern

Methods

n/a	Involved in the study	
☒	☐	ChIP-seq
☐	☒	Flow cytometry
☒	☐	MRI-based neuroimaging

Antibodies

Antibodies used	Anti-SARS-CoV-2 Neutralizing human IgG1 Antibody from Acro Biosystems, NAb#1 (Fig 4c, d) Cat#: SAD-S35<br>GenScript clone ID 6D11F2, NAb#2 (Fig 4d) Cat#: A02055<br>GenScript clone ID 10G6H5, NAb#3 (Fig 4d)<br>Invitrogen clone ID MA5-35939 Nab#4 (Fig 4d) Cat#: MA5-35939
Validation	No validation statements are currently available for the antibodies used.

Eukaryotic cell lines

Policy information about cell lines

Cell line source(s)	293T (ATCC CRL-11268)<br>293TAce2 (derived from 293T); new cell line generated in this study
Authentication	Cell lines were authenticated by the source.
Mycoplasma contamination	The cells were checked for mycoplasma contamination with PlasmoTest Mycoplasma detection kit from Invivogen.
Commonly misidentified lines (See ICLAC register)	No commonly misidentified cell lines were used.

Human research participants

Policy information about studies involving human research participants

Population characteristics	We enrolled 69 female and 46 male COVID-19 subjects with an average age of 54 and 51, respectively.
Recruitment	The subjects (n=115) were recruited at SUNY Downstate Medical Center, New York, NY, Cedars Sinai, Los Angeles, CA, or the University of Connecticut, School of Medicine, Farmington CT following testing and/or admission for COVID-19 infection. Written informed consent was obtained from all participants in this study and was approved by the following IRBs: 1) IRB# SUNY:269846. The patients were recruited at SUNY Downstate, NY and processed and biobanked at Amerimmune, Fairfax VA; 2) IRB# STUDY00000640. Convalescent plasma was collected at Cedars Sinai Medical Center according to FDA protocol (https://www.fda.gov/vaccines-blood-biologics/investigational-new-drug-ind-or-device-exemption-ide-process-cber/recommendations-investigational-covid-19-convalescent-plasma#Collection%20of%20COVID-19). The source of the convalescent plasma was volunteer blood donors who were recovered from COVID-19. Donors met routine blood donor eligibility requirements established by the FDA and had a prior SARS-CoV-2 infection documented by a laboratory test for the virus during illness, or antibodies to the virus after recovery of suspected disease. All donors were at least 28 days from either resolution of COVID-19 symptoms or diagnostic clearance, whichever was longer; 3) IRB# 20-186-1. UConn Healthcare workers who tested positive for the virus by PCR were recruited and samples banked for future testing. 4) IRB#: 17-JGM-13-JGM or 16-JGM-06-JGM. De-identified control subjects (n=56) with previously frozen (more than a year ago) samples obtained from healthy controls or determined to be SARS-CoV-2 PCR negative (IRB SUNY:269846). All antibody assays were performed at the Jackson Laboratory for Genomic Medicine, Farmington, CT. Subject characteristics are shown in Table 1.
Ethics oversight	See above

Note that full information on the approval of the study protocol must also be provided in the manuscript.

Flow Cytometry

Plots

Confirm that:

☒ The axis labels state the marker and fluorochrome used (e.g. CD4-FITC).

☒ The axis scales are clearly visible. Include numbers along axes only for bottom left plot of group (a 'group' is an analysis of identical markers).

☒ All plots are contour plots with outliers or pseudocolor plots.

☒ A numerical value for number of cells or percentage (with statistics) is provided.

Methodology

Sample preparation	All plasma samples for antibody assays were aliquoted and stored at -80°C. Prior to experiments, aliquots of plasma samples were heat-inactivated at 56°C for 30 minutes.<br>HEK293 cells for neutralization assays were cultured in complete RPMI 1640 medium (RPMI 1640 supplemented with 10% FBS; Atlanta Biologicals, Lawrenceville, GA), 8% GlutaMAX (Life Technologies), 8% sodium pyruvate, 8% MEM vitamins, 8% MEM nonessential amino acid, and 1% penicillin/streptomycin (all from Corning Cellgro) for 72 hours, collected using %0.05 Trypsin-0.53 mM EDTA (Corning Cellgro) for flow cytometry.
Instrument	Samples were acquired on a BD FACSymphony A5 analyzer
Software	Data were analyzed using FlowJo (Tree Star).
Cell population abundance	This is not relevant as no sorting were performed in this study
Gating strategy	Gating strategy for Flow immunoassay:<br>DevScreen SAv Beads were gated using FSC-H/SSC-H, and singlet beads gate was created using FSC-A/FSC-H. Gates for different DevScreen SAv Beads were determined based on their fluorescence signature on RL1-H/RL2-H plot (on iQue plus). PE fluorescence median, which is directly associated with each single plex beads was determined using BL2-H (on iQue plus).<br>Gating strategy for neutralization assay:<br>Total cells were gated using FSC-A/SSC-A, GFP and RFP cell populations were gated using GFP-A/PE-A (BD FACSymphony A5 analyzer). Cells that do not express GFP or RFP were used to define the boundaries between positive and negative cell populations. To report the percent infection in ACE2 positive cells, total cells were sub-gated on GFP+ and RFP+ cell population was determined using PE-A.

☐ Tick this box to confirm that a figure exemplifying the gating strategy is provided in the Supplementary Information.
